# Target-selective joint polymerase chain reaction: A robust and rapid method for high-throughput production of recombinant monoclonal antibodies from single cells

**DOI:** 10.1186/1472-6750-11-75

**Published:** 2011-07-21

**Authors:** Megumi Yoshioka, Nobuyuki Kurosawa, Masaharu Isobe

**Affiliations:** 1 Graduate School of Innovative Life Science, University of Toyama, Toyama-shi, Toyama, 930-8555, Japan; 2 Laboratory of Molecular and Cellular Biology, Faculty of Science and Engineering, Graduate School, University of Toyama, 3190 Gofuku, Toyama-shi, Toyama, 930-8555, Japan; 3 Laboratory of Molecular and Cellular Biology Faculty of Science and Engineering, Graduate School, University of Toyama, 3190 Gofuku, Toyama-shi, Toyama, 930-8555, Japan

## Abstract

**Background:**

During the development of a therapeutic antibody, large numbers of monoclonal antibodies are required to screen for those that are best suited for the desired activity. Although the single cell-based immunoglobulin variable gene cloning technique is a powerful tool, the current methods remain an obstacle to the rapid production of large numbers of recombinant antibodies.

**Results:**

We have developed a novel overlap extension polymerase chain reaction, the target-selective joint polymerase chain reaction (TS-jPCR), and applied it to the generation of linear immunoglobulin gene expression constructs. TS-jPCR is conducted using a PCR-amplified immunoglobulin variable gene and an immunoglobulin gene-selective cassette (Ig-cassette) that contains all essential elements for antibody expression and overlapping areas of immunoglobulin gene-specific homology. The TS-jPCR technique is simple and specific; the 3'-random nucleotide-tailed immunoglobulin variable gene fragment and the Ig-cassette are assembled into a linear immunoglobulin expression construct, even in the presence of nonspecifically amplified DNA. We also developed a robotic magnetic beads handling instrument for single cell-based cDNA synthesis to amplify immunoglobulin variable genes by rapid amplification of 5' cDNA ends PCR. Using these methods, we were able to produce recombinant monoclonal antibodies from large numbers of single plasma cells within four days.

**Conclusion:**

Our system reduces the burden of antibody discovery and engineering by rapidly producing large numbers of recombinant monoclonal antibodies in a short period of time.

## Background

Recombinant monoclonal antibody technology comprises a series of molecular approaches that allows for the production of therapeutic antibodies [1,2]. Molecular cloning and expression of polymerase chain reaction (PCR)-amplified immunoglobulin variable (V) genes from single, isolated primary B cells provide powerful tools for the generation of recombinant monoclonal antibodies [3,4]. Introduction of the PCR-amplified V gene fragments into expression plasmids has been performed using traditional cut-and-paste DNA cloning techniques [5-9]. Recently, site-specific recombination and homologous recombination cloning techniques, which eliminate the use of restriction endonucleases and ligases, offer several advantages in the context of high-throughput procedures [10-14]. These methods, however, still require plasmid amplification in bacteria, followed by plasmid purification and verification of the insert.

Because of the need for a more convenient method for the generation of recombinant antibodies, the overlap extension polymerase chain reaction method (overlap PCR) has been developed. In this method, a PCR-amplified V gene fragment is joined to DNA cassettes by PCR to build a linear immunoglobulin gene expression (Ig-expression) construct [15-17]. While the current overlap PCR method is rapid compared with traditional plasmid-based cloning methods, it still has several limitations. One of the major drawbacks of this method is that the PCR-amplified V gene fragment must be purified to remove primers and nonspecifically amplified DNA fragments to achieve successful production of Ig-expression constructs. Because short homology overlaps within the ends of DNA cassettes are generated at the ends of PCR-amplified DNA fragments with primers, both V gene fragments and nonspecifically amplified PCR products are joined to the DNA cassettes. Another problem is this technique's complicated joining reaction in which a promoter cassette, the purified V gene fragment and a terminator cassette must be assembled in a specific order based on their short homology overlaps. This process sometimes results in a low yield of Ig-expression constructs. Therefore, a more efficient system that bypasses these tedious steps is required to generate recombinant antibodies from large numbers of single, isolated cells.

This study describes a novel overlap PCR method termed target-selective joint PCR (TS-jPCR). With this method, a PCR-amplified V gene fragment can be selectively assembled into a linear Ig-expression construct, even in the presence of nonspecifically amplified DNA fragments. TS-jPCR is accomplished by joining the 3'-random nucleotide-tailed V gene fragment and an immunoglobulin-selective cassette (Ig-cassette). The Ig-cassette contains all the essential elements for antibody expression and V-gene-specific long homology overlaps within a single DNA molecule.

**Figure 1:**
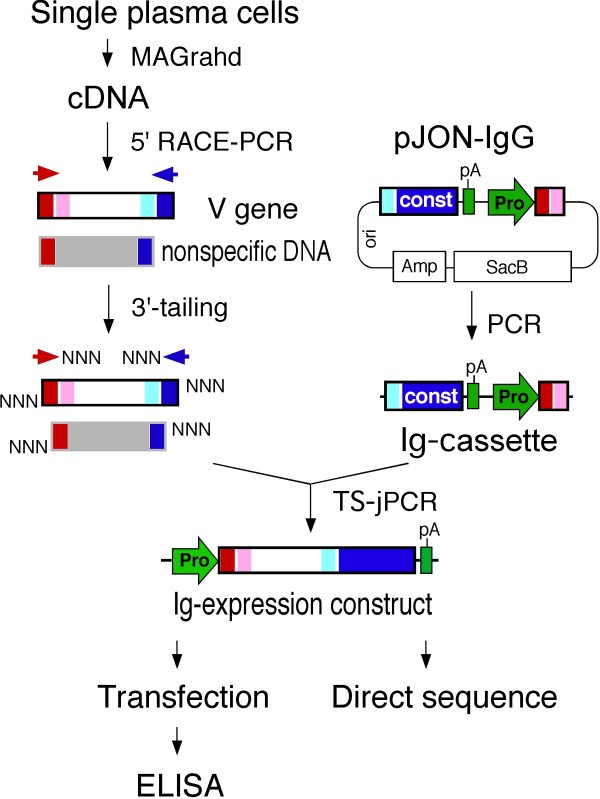
**A flow chart summarizing the high-throughput production of recombinant antibodies from single plasma cells**. Single cell-based cDNA synthesis was performed by MAGrahd. V genes were amplified from the cDNA by 5'-RACE PCR (Day 1). The PCR products were treated with TdT for 3'-end random nucleotide tailing. The reaction products were then mixed with Ig-cassettes to generate linear Ig-expression constructs by TS-jPCR. Cognate pairs of IgH- and IgL-expression constructs were then directly transfected into 293FT cells (Day 2). The V-(D)-J repertoire and IgG subclass were determined by direct sequencing (Days 3-4). The concentration and activity of the recombinant antibodies were determined by ELISA (Day 4). Pro, promoter; pA, poly(A) site; const, immunoglobulin constant region; NNN, 3'-end random-nucleotide tail.

We also developed a robotic magnetic head handling instrument (MAGrahder) that allows for automated single cell-based cDNA synthesis and 3' end homopolymer tailing using the MAGrahd method [18]. The MAGrahder is a non-contact magnetic power transmission instrument in which 12-channel, parallel magnetic rods installed on a robotic arm transport nucleic acid-bound magnetic beads in a MAGrahd reactor tray. Using MAGrahder and TS-jPCR, we were able to produce recombinant monoclonal antibodies from large numbers of single plasma cells within four days (Figure 1).

## Results

### Development of TS-jPCR

**Figure 2:**
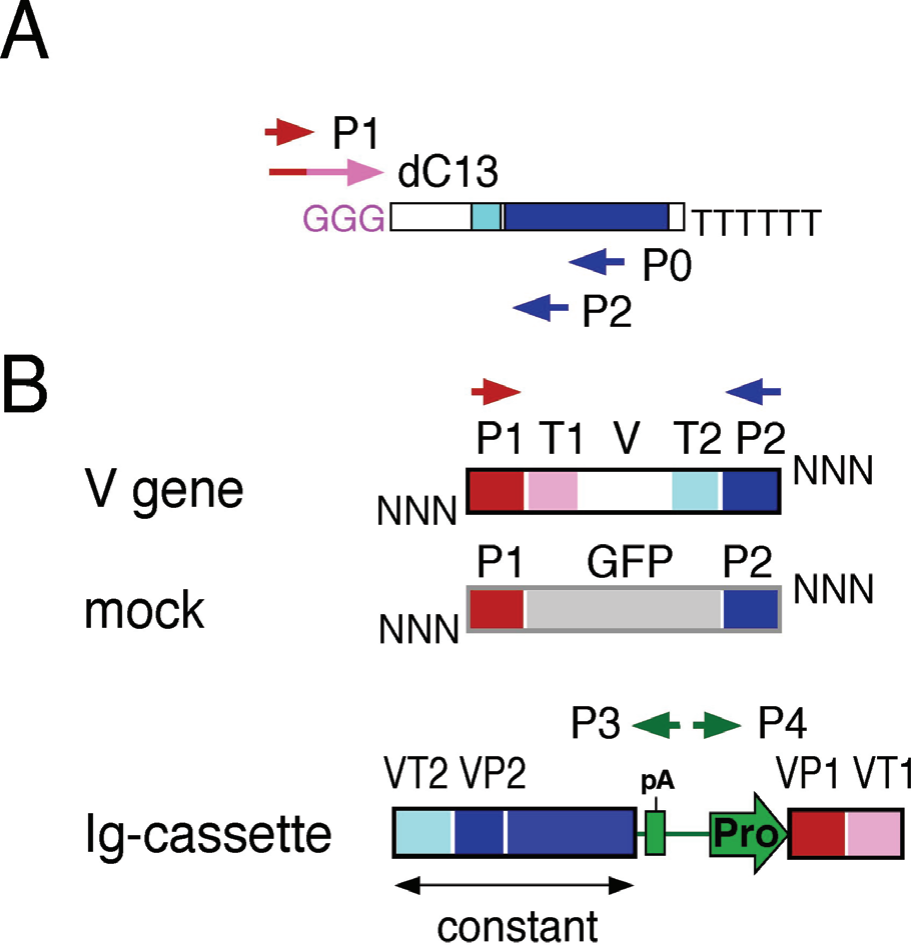
**Schematic illustration of DNA fragments and primer positions**. (A) 3'-end poly dG-tailed cDNA and primers used for the amplification of V genes by 5'-RACE PCR. Arrows represent the position and orientation of the primers. dC13 is the first-round PCR forward primer specific for the poly dG-tailed cDNA, and P0 is the first-round PCR reverse primer specific for the respective IgG or IgK constant regions. P1 is the second round PCR forward primer, and P2 is the second round PCR reverse primer specific for the IgG or IgK constant regions. (B) Schematic representation of homology overlaps between the Ig-cassette and DNA fragments. The Ig-cassette contains V gene-specific sequences (VT1 and VT2) external to the primer-derived sequences (VP1 and VP2). The V gene contains specific sequences (T1 and T2) for the 5'-RACE PCR-amplified V gene internal to the primer-derived sequences (P1 and P2). The mock DNA has P1 and P2 sequences at its ends. P3 and P4 PCR primers hybridize to the f1 replication origin of the Ig-cassette and are used for amplifying the Ig-expression constructs.

**Figure 3:**
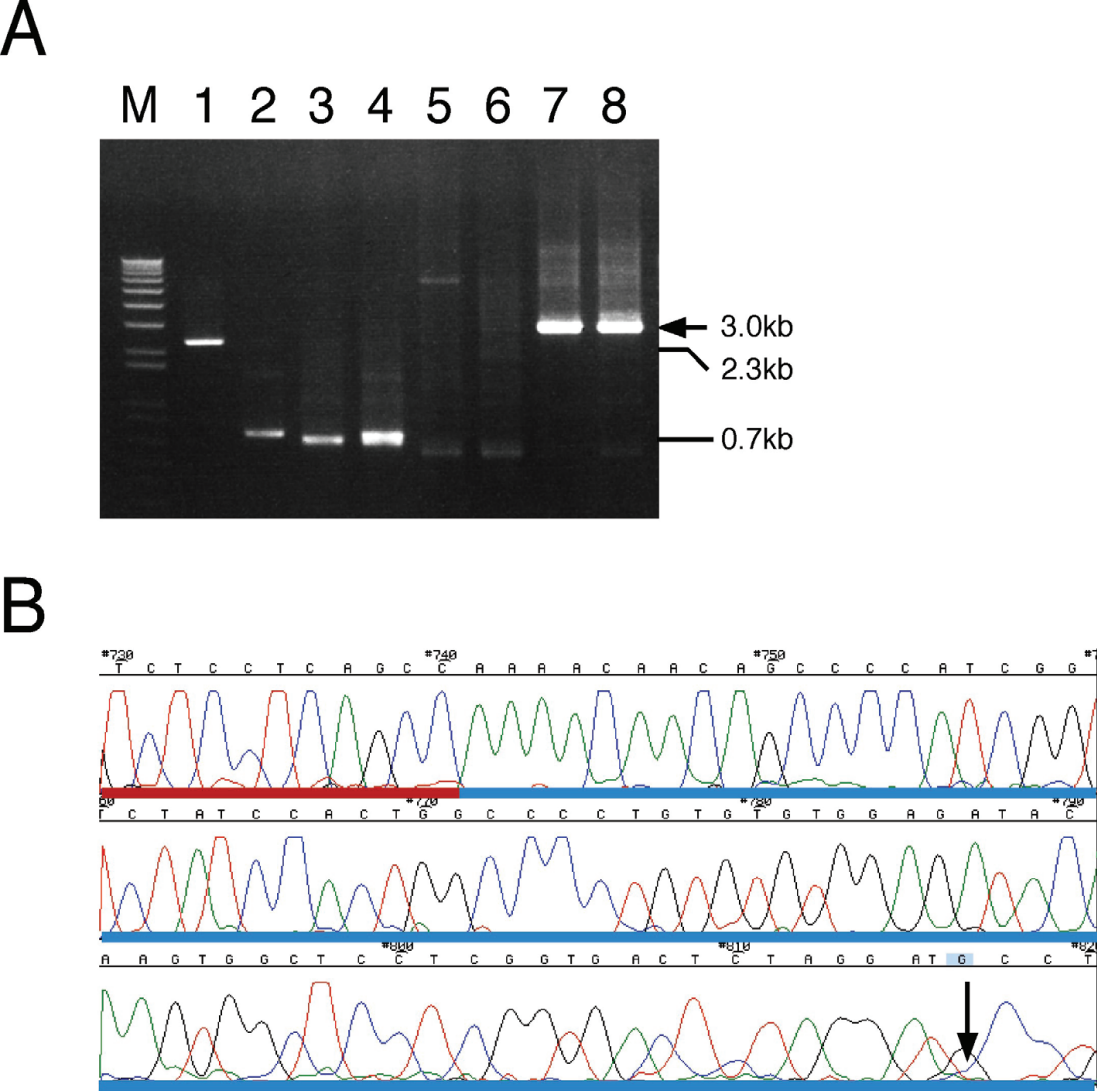
**Pilot experiment showing the selective joining of a V gene to the Ig-cassette by TS-jPCR**. (A) An agarose gel stained with ethidium bromide showing the TS-jPCR results. Lane 1: Ig-cassette, lane 2: tailed mock DNA fragment, lane 3: tailed V gene fragment, lane 4: mixture of the mock and V gene fragments, lane 5: amplified product with the Ig-cassette and water, lane 6: amplified fragment with the Ig-cassette and mock DNA fragment, lane 7: amplified fragment with the Ig-cassette and V gene fragment, lane 8: amplified fragment with the Ig-cassette and a mixture of mock DNA and V gene fragments. Lanes 5-8: each lane contains 1 μl from a 25-μl PCR. The arrow indicates the size of the Ig-expression construct. M, 1 kb DNA ladder. (B) DNA sequence chromatogram of the fragment in lane 8. The arrow indicates the boundary between the V gene and the Ig-cassette. The blue horizontal bar indicates the heavy chain constant sequence, and the red bar indicates the variable sequence.

To evaluate the performance of TS-jPCR, we conducted a pilot experiment using an artificially amplified mouse V gene and a mock DNA fragment (Figure 2). The mock DNA fragment served as a model for a nonspecifically amplified DNA fragment and was composed of an upper PCR primer (P1), a green fluorescent protein (GFP) gene segment and a lower PCR primer (P2) sequence. The V gene fragment was composed of the P1 sequence, a poly dG/C sequence (T1), a mouse immunoglobulin heavy chain variable sequence, part of the constant gene sequence (T2) and the P2 sequence. The T1 and T2 sequences are specific regions of V gene fragment amplified by the rapid amplification of 5' cDNA ends PCR (5'-RACE PCR). An Ig-cassette contains all essential elements for expression of the antibody, including the CMV promoter, the immunoglobulin chain constant region and the poly (A) signal. Additionally, the cassette has long overlapping regions of immunoglobulin gene-specific homology on its ends (VP1VT1 and VP2VT2). VT1 (VT2) sequence localizes at the end of the Ig-cassette and shares homology with T1 (T2) sequence. VP1 (VP2) sequence localizes internally to the VT1 (VT2) sequence and shares homology with P1 (P2) sequence. Before the joining of these DNA fragments to the Ig-cassette, each DNA fragment was tailed with random nucleotides on its 3' end using a terminal deoxynucleotidyl transferase (TdT). As shown in Figure 3, the joining of the tailed V gene fragment to the Ig-cassette by TS-jPCR resulted in the amplification of a single major band corresponding to the expected size of the linear Ig-expression construct. When TS-jPCR was conducted with the tailed mock DNA fragment and the Ig-cassette, no amplification product was detected. When TS-jPCR was conducted with the cassette and a mixture of the V gene and the mock DNA fragment (1:1 molar ratio), a single major band corresponding to the expected size was detected (Figure 3A). To examine whether the amplified band resulted from the Ig-cassette joining to the V gene, the mock or both, we performed direct sequencing. As shown in Figure 3B, the sequencing showed a clear chromatograph pattern that corresponded to the V gene sequence. Furthermore, no insertion or deletion at the joint junctions was found. These results clearly confirm the high selectivity of the TS-jPCR method, even in a case in which the nonspecific amplification happened to be the same size as the V gene fragment.

**Figure 4:**
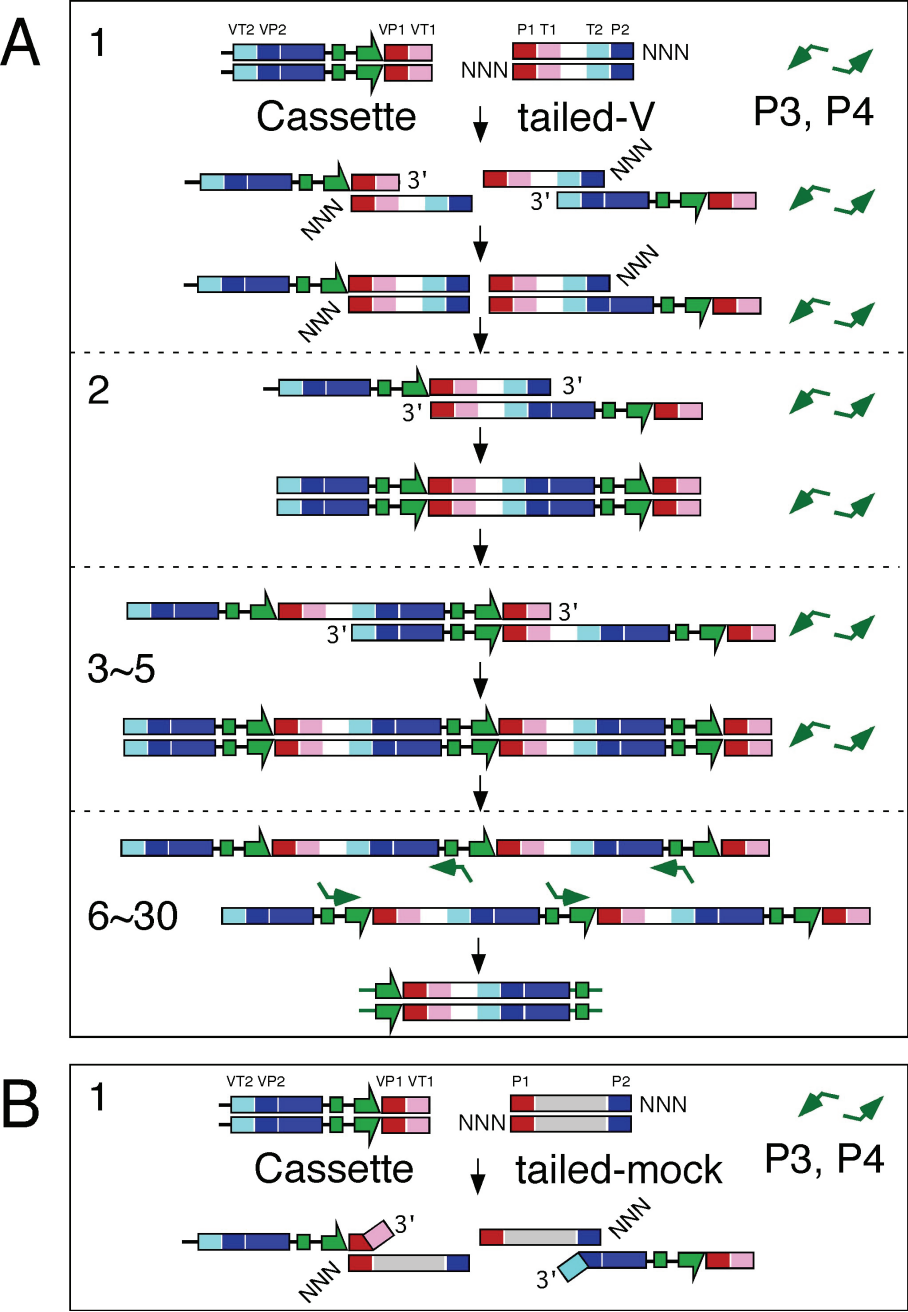
**Schematic illustration of TS-jPCR**. (A) Diagram showing the specific V gene joining with the Ig-cassette. (B) Diagram showing the mechanism by which TS-jPCR inhibits the joining of nonspecifically amplified DNA to the Ig-cassette. P3 and P4 are primers that hybridize to the f1 replication origin sequence localized between the CMV promoter and the poly (A) signal under a low annealing temperature. NNN indicates the 3'-end random-nucleotide tail.

The scheme in Figure 4 explains the mechanism that enables the selective joining of TS-jPCR. When TS-jPCR is conducted, the melted strands of the Ig-cassette and the V gene fragment partially hybridize with each other via the long homology overlaps (P1T1 to VP1VT1 and P2T2 to VP2VT2) to generate stable hybrid duplexes. Each of the hybridized cassette strands that contain the V gene-specific sequence at the 3' ends (VT1 or VT2) can serve as a primer for strand extension, which leads to the generation of an Ig-cassette-V gene fusion strand in each hybrid duplex. However, each of the hybridized V strands that contain 3'-end random nucleotide tails fails to serve as a primer (1st PCR cycle). After denaturing the hybrid duplexes, the melted fusion strands partially hybridize with each other via the complementary V gene sequences, which leads to the generation of a double-strand DNA that is composed of two copies of the Ig-cassette joined to each end of the V gene in a head-to-tail orientation (2nd PCR cycle). This repeating unit facilitates partial hybridization of each strand, which leads to the generation of an Ig-cassette-V gene concatemer (3rd to 5th PCR cycle). Until the end of the fifth PCR cycle, the P3 and P4 PCR primers used for amplification of an Ig-expression construct do not hybridize with their complementary strands because of the high annealing temperature. Conducting the following 30 additional PCR cycles at a low annealing temperature allows the P3 and P4 PCR primers to hybridize with their complementary strands, which leads to amplification of the Ig-construct.

When TS-jPCR is conducted with the Ig-cassette and the tailed mock DNA, the melted strands of the Ig-cassette and mock DNA partially hybridize with each other via their short homology overlaps (P1 to VP1 and P2 to VP2) to generate unstable hybrid duplexes. It is important to note that these structures render the polymerase unable to extend any strands because of the 3'-end mismatches. Therefore, nonspecifically amplified DNA that lacks the V gene-specific sequence (T1 and T2) internal to the primer-derived sequence (P1 and P2) is unable to be joined to the Ig-cassette (Figure 4B).

### High-throughput amplification of immunoglobulin variable genes

**Figure 5:**
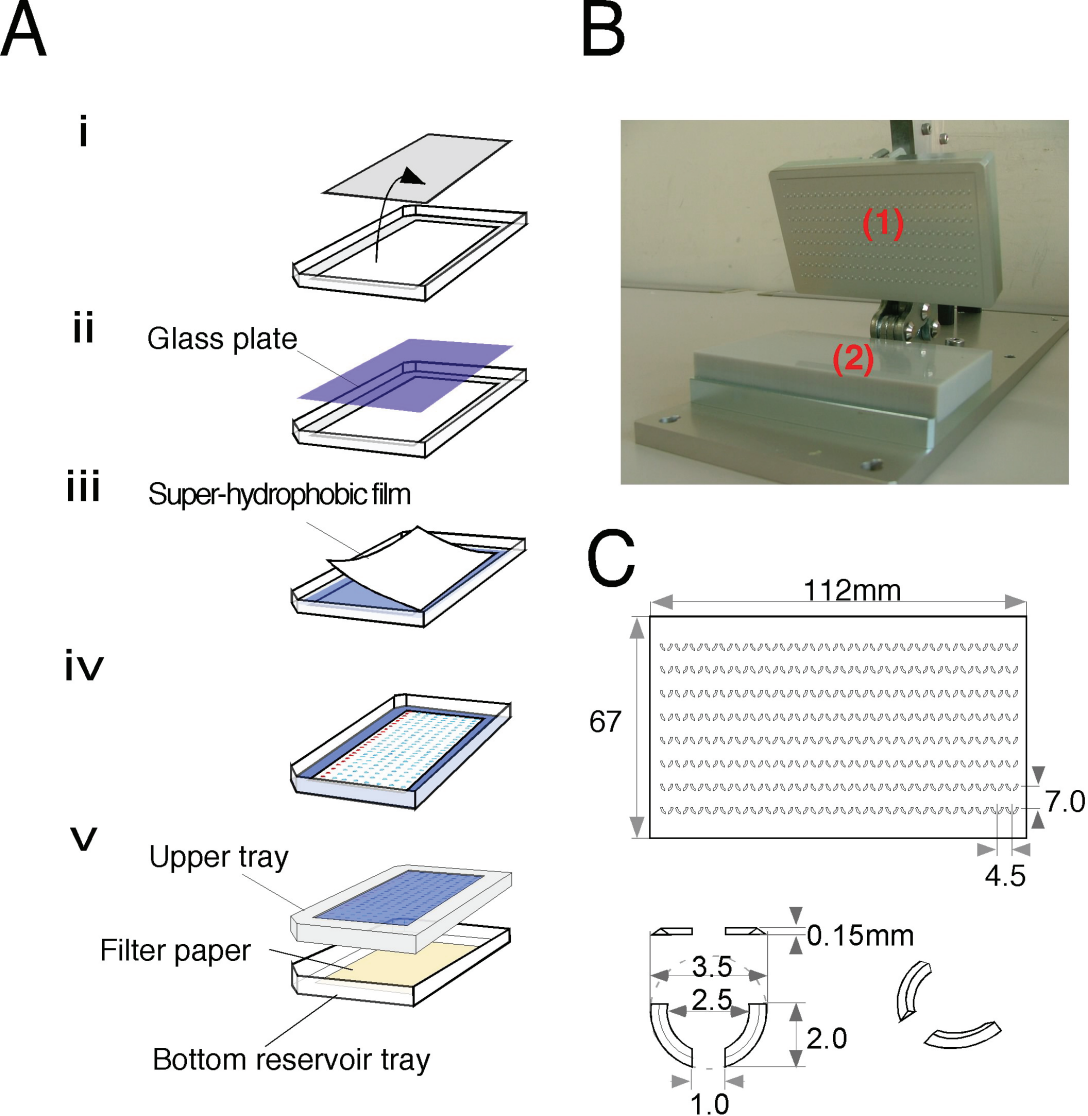
**Design and assembly of the MAGrahd reactor tray**. (A) Flow diagram for the set-up of the MAGrahd device.(i) The upper tray plate of an Omni tray (Nunc) was cut into a rectangular shape (49 mm × 110 mm). (ii) The opening was fixed with a cover glass plate from the inside of the lid. (iii) The fabricated film was bound to the inner surface of the thin glass plate. (iv) All of the reagents were dispensed onto the grids of the super-hydrophobic film. (v) The upper tray was inverted and attached to the bottom reservoir tray. (B) Design of the master stamp for embossing an array of grids onto a sheet of super-hydrophobic film. (1) An aluminum master and (2) a silicone plate. (C) Schematic drawing of an array of grids on a super-hydrophobic film.

**Figure 6:**
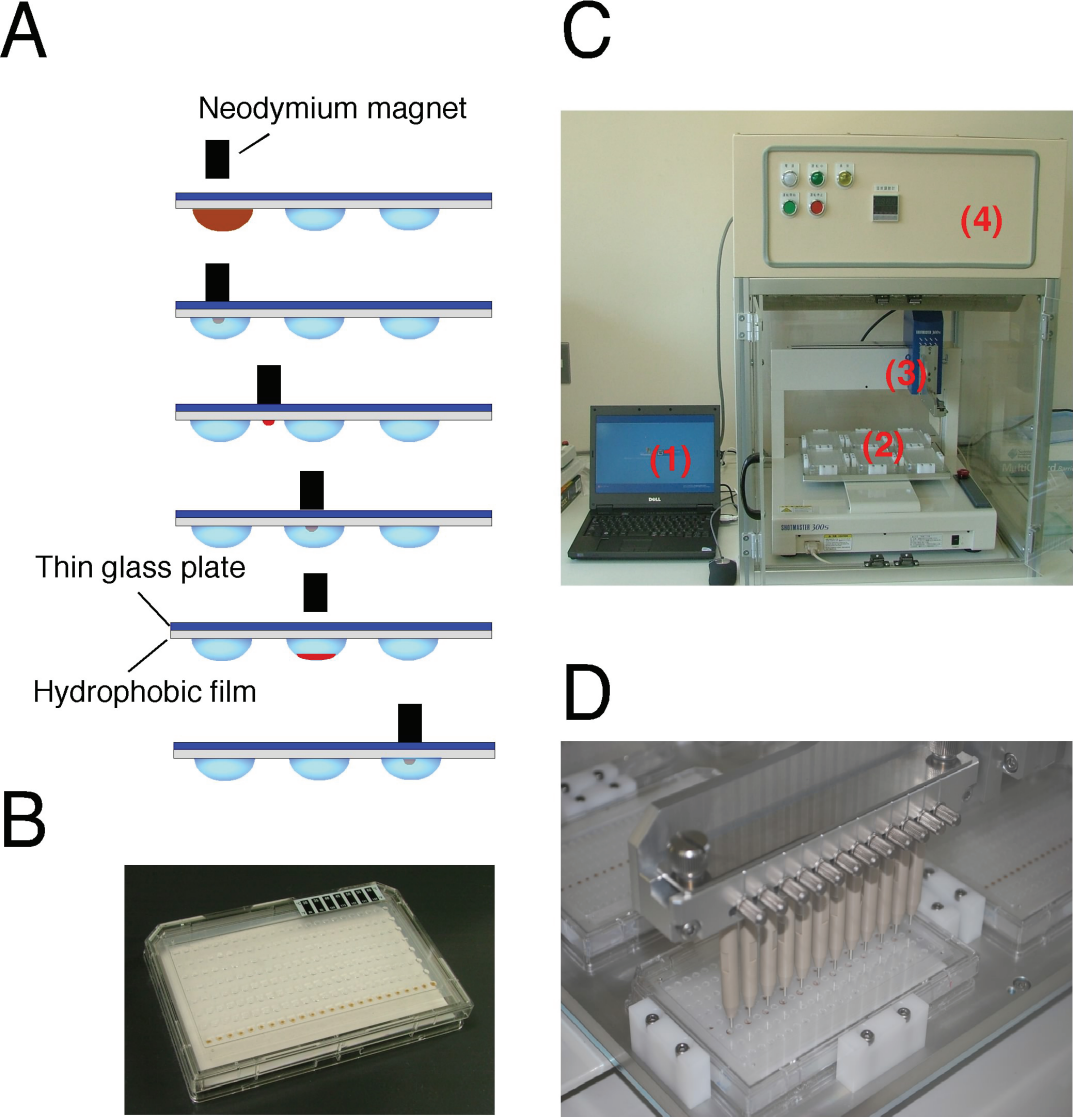
**Design of robotic magnetic bead handling instrument (MAGrahder)**. (A) A lateral view shows how magnetic beads are extracted from a drop, transferred into the next drop and mixed by MAGrahder. When the neodymium permanent magnet rod contacts the thin glass surface of the MAGrahd reactor tray from outside near the drops, the magnetic beads in the drops collect at the surface of the glass plate. The beads are then transported to subsequent drops where they are released by removing the magnetic rod from the glass plate. (B) A lateral view of the MAGrahd tray reactor after set-up. (C) General view of the MAGrahder. (1) Computer control; (2) the deck of the ShotMaster 300 that is equipped with six MAGrahd reactor trays; (3) robotic arm; (4) temperature controller. (D) Image of the robotic arm with 12-channel magnetic rods transferring magnetic beads in the MAGrahd reactor tray.

**Figure 7:**
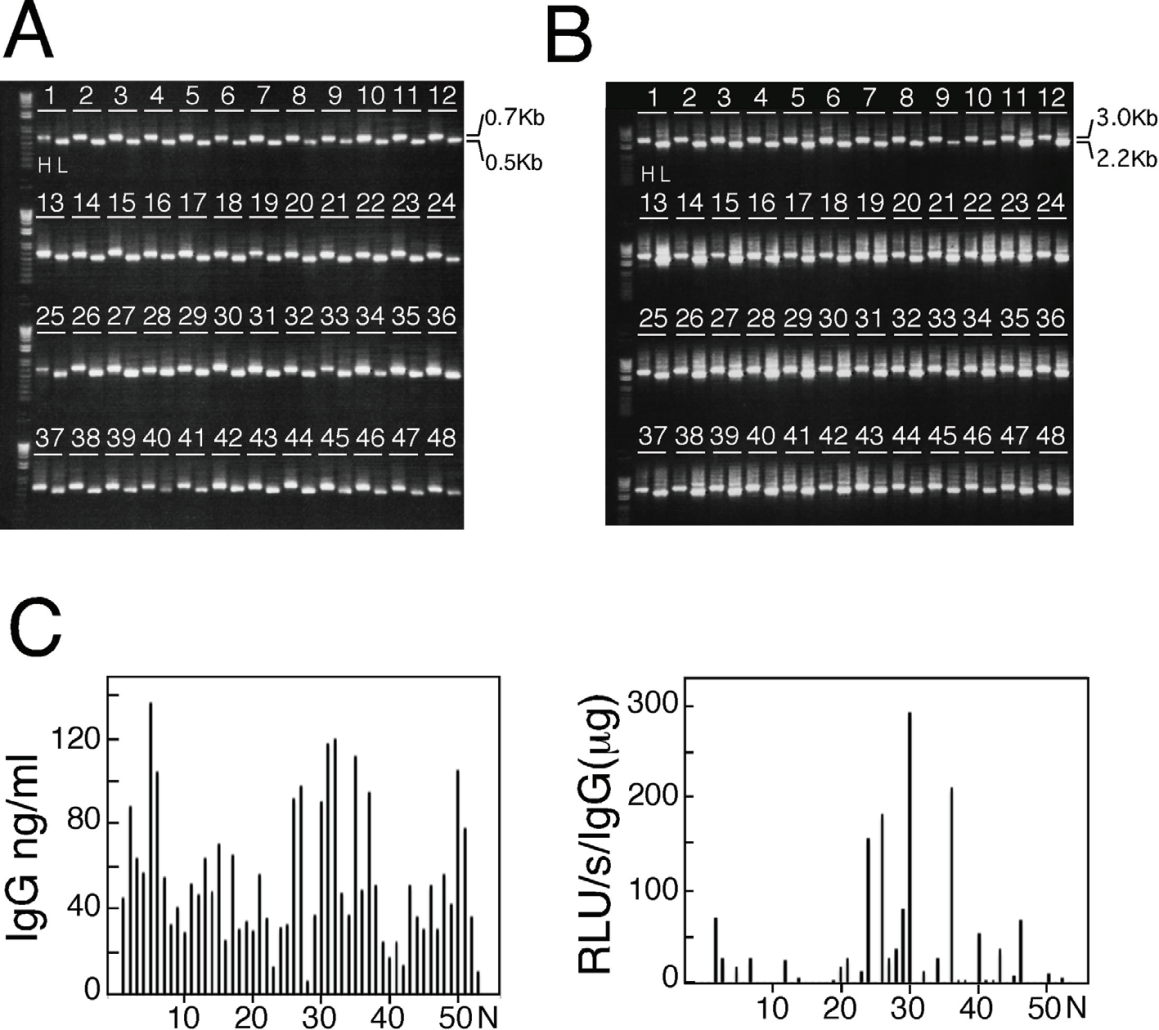
**High-throughput production of IgH- and IgL-expression constructs by TS-jPCR**. (A) Ethidium bromide-stained agarose gels of the cognate pairs of VH and VL genes amplified from single plasma cells. Each lane contains 1 μl of a 25-μl VH or VL 5'-RACE PCR product. Representative PCR products from fifty-three samples are shown. H, heavy chain V gene fragment; L, light chain V gene fragment. (B) Agarose gels stained with ethidium bromide showing the TS-jPCR results. VH and VL genes obtained in (A) were treated with TdT and joined to the corresponding Ig-cassette. Each lane contains 1 μl of a 25 μl IgH- or IgL-expression construct. The fainter high-molecular weight bands are presumed to be a multimer of the correct band. Representative PCR products from fifty-three samples are shown. H, IgH-expression construct; L, IgL-expression construct. (C) Production of recombinant mouse monoclonal antibodies. Cognate pairs of IgH- and IgL-expression constructs (1 μl each) were directly transfected into 293FT cells. The concentration of the recombinant antibodies in the cell culture media was determined by a sandwich ELISA two days after the transfection (left panel). Antigen specificity against egg albumin is expressed as luminescence activity (right panel). The specific activity of recombinant antibodies is expressed as relative light units (RLU/s/IgG (μg)). N indicates a negative control; all incubation steps were identical except that nontransfected cell culture medium was used.

Single cell-based cDNA synthesis is a costly and time-consuming process for the production of recombinant monoclonal antibodies from large numbers of single cells. To speed up this process, we developed an automatic non-contact magnetic power transmission instrument, MAGrahder. MAGrahder is composed of a MAGrahd reactor tray and a desktop robot. The MAGrahd reactor tray can be assembled from a reusable tray and a single-use super-hydrophobic film upon which a 24 × 8 grid of cup-shaped structures to hold drops are embossed (Figure 5). The robot provides a tip- and tube-free platform that allows magnetic beads to be transferred between arrayed hanging drops deposited on the cup-shaped structures of the super-hydrophobic film with an externally applied magnetic force. MAGrahder has 12-channel, parallel magnetic rods installed on a robotic arm, which transports and mixes nucleic acid-bound magnetic beads in MAGrahd reactor trays (Figure 6). The instrument performs mRNA extraction, reverse transcription and the homopolymer-tailing reaction and is capable of handling up to 144 samples per instrument, in parallel, within an hour by touching neodymium permanent magnet rods to the opposite side of the thin glass surface of the MAGrahd reactor tray above the drops. We prepared 3'-end homopolymer-tailed cDNA from isolated single plasma cells and used them as templates to amplify immunoglobulin heavy chain variable (VH) and immunoglobulin light chain variable (VL) genes by 5'-RACE PCR. As shown in Figure 7A, 5'-RACE PCR resulted in the successful amplification of the cognate pair of VH and VL genes with an overall success rate of 100%.

### Application of TS-jPCR for the production of recombinant mouse antibodies

**Figure 8:**
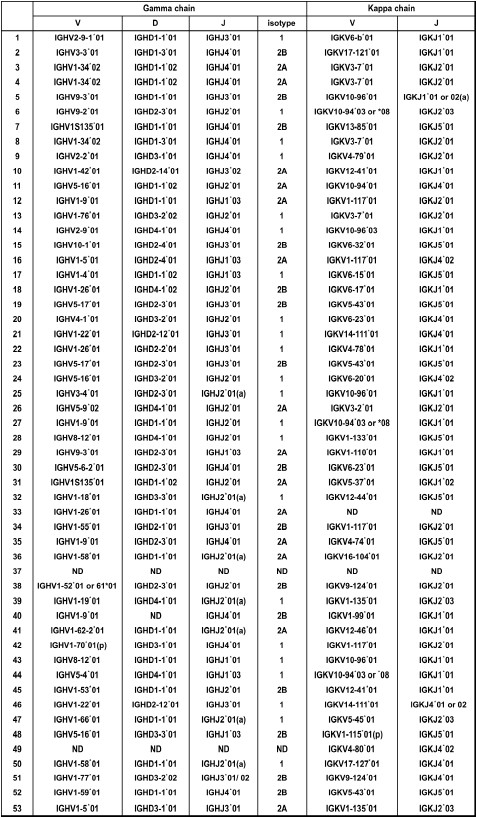
**V-(D)-J repertoire of the assembled V genes**. Linear IgH- and IgK-expression constructs were directly sequenced, and the V-(D)-J-(H) repertoires were determined. The sequence primers used were P2.

We next attempted to demonstrate the utility of TS-jPCR by producing Ig-expression constructs from amplified V genes. After 3'-end random nucleotide tailing of the 5'-RACE PCR products, they were joined to the respective Ig-cassette using TS-jPCR. As shown in Figure 7B, TS-jPCR resulted in the amplification of single major bands corresponding to the expected sizes of IgG- and IgK-expression constructs. The joining success rate was 100% in both IgG and IgK. When we transfected the cognate pair of unpurified Ig-expression constructs into 293FT cells, expression of the recombinant monoclonal antibodies was detected in all examined samples (Figure 7C). Analysis of the antigen binding activity of the recombinant antibodies revealed that eight clones specifically reacted with the antigen (Figure 7D). Because of the high selectivity of TS-jPCR, we next attempted to determine the V-(D)-J repertoire of the assembled V genes. Direct sequencing of the Ig-expression constructs was readily achieved with an overall sequencing success rate of 96% (Figure 8). The sequencing failed in only two cases of VH (37 and 49) and two cases of VL (33 and 37). Cloning of the corresponding 5'-RACE PCR products into plasmids revealed that these fragments contained pseudo V gene segments that had T1 and T2 sequences internal to the P1 and P2 sequences.

## Discussion

We have established TS-jPCR and used it for the rapid production of recombinant monoclonal antibodies from mouse plasma cells. While the principle of this procedure is essentially the same as other recently reported PCR-based methods, TS-jPCR has several useful features that overcome the limitations of the current methods. First, purification of the amplified V genes represents a significant, major bottleneck for the high-throughput production of recombinant antibodies. In contrast, TS-jPCR enables the production of Ig-expression constructs, even in cases where the nonspecific amplification product happens to be the same size as the V genes. Second, current PCR-based methods sometimes suffer low Ig-expression construct yields. This result is likely due to inefficient hybrid duplex formation between the V gene and the promoter or terminator cassette, which is mediated through their short homology overlaps (~25 bp). TS-jPCR can produce a sufficient amount of linear expression construct for DNA transfection and direct sequencing by using a single cassette that has long and specific homology overlaps (over 50 bp) at both ends.

Several methods for single cell-based cDNA synthesis have been reported to amplify V genes by 5'-RACE PCR, including the use of multi-well methods [19]. However, these methods generally require manual input, thereby limiting the throughput of the experiment and increasing the susceptibility to cross-contamination and nonspecific amplification. The MAGrahder provides more effective single cell-based cDNA synthesis by using a non-contact automatic magnetic power transmission system in which the magnetic beads are transported and mixed by the combined action of magnetic and gravitational fields in the hanging drop. The advantage of MAGrahd is that high quality cDNA synthesis for downstream applications can be achieved in a short time because nucleic acid-bound magnetic beads were separated from residual contaminants, such as salts, nucleotides and enzymes, by extracting the beads from the drops. Additionally, the MAGrahd reactor tray protects against cross-contamination because all steps were proceed in a pipet tip-free environment in a closed space. Furthermore, the technical variation is significantly decreased when compared to a corresponding manual experiment, which results in the successful amplification of V genes by 5'-RACE PCR. The fact that the gene amplification unit is not integrated may be considered a disadvantage of MAGrahder. However, the collection of cDNAs from the MAGrahd reactor tray can be easily transferred into the wells of a 96- or 384-well PCR plate with a twelve-tipped pipettor. Given the time required for PCR, it is far more efficient for the machine to solely operate for cDNA synthesis. Thus, there is no need to include the gene amplification unit into MAGrahder.

We recently developed a target-selective homologous recombination cloning method (TS-HR) for the single-step insertion of PCR-amplified V gene fragments into expression vectors [18]. Because TS-HR is a plasmid-based cloning method, TS-jPCR is preferable to TS-HR in the context of high-throughput production of large numbers of recombinant antibodies. Once recombinant monoclonal antibodies with the desired specificity have been obtained by screening, the corresponding V genes can be inserted into an expression plasmid by TS-HR, which can in turn be used for large-scale antibody production.

## Conclusion

Starting from fifty-three isolated mouse plasma cells, we achieved amplification of the VH and VL genes within a single day. On the next day, each of the amplified V genes was joined to an Ig-cassette by TS-jPCR to generate Ig-expression constructs that can be used for DNA transfection and direct sequencing. Our methods are directly applicable to rapid and scalable automation for the generation of large numbers of recombinant monoclonal antibodies.

## Methods

### Materials

Dynabeads oligo(dT)25 magnetic beads, Dulbecco's Modified Eagle's Medium, SuperScript III Reverse Transcriptase and One-shot TOP10 competent cells were obtained from Invitrogen. Super-hydrophobic film was obtained from Alcan Packaging. The FuGENE^® ^HD Transfection Reagent and TdT were obtained from Roche Applied Science. PrimeSTAR™ HS DNA Polymerase was obtained from Takara Bio. Restriction enzymes were obtained from New England Biolabs. Titermax Gold Adjuvant was obtained from CytRx Corporation. A SHOTMASTER 300 desktop robot was purchased from Musashi Engineering. The animal experiment was approved by the Committee on Animal Experimentation at Toyama University.

### Plasmid construction

A DNA fragment encoding 901 bp of the mouse immunoglobulin gamma (IgG) constant region (559-1460 of nucleotide accession number: AB097849) was amplified with the primers 5'-- GATATCACGTGTGCCTGGTCAAGGGCTATTTCCCTGAG-3' and 5'-- CTCCGCGGCCGCTGGGATCATTTACCAGGAGAGT -3' (restriction sites underlined). A DNA fragment encoding 309 bp of the mouse immunoglobulin kappa (IgK) constant region (441-750 of nucleotide accession number: AF466770) was amplified with the primers 5'--GATATCACGTGCTGTATCCATCTTCCCACCATCC -3' and 5'--TCTCGCGGCCGCTGTCTCTAACACTCATTCCTG-3'. A DNA fragment containing the pUC replication origin, an ampicillin resistance gene and a SacB gene was amplified from pDNR (Takara-Clontech) by PCR with the primers 5'-AGAGAGACCCGGGCCAGGAACCGTAAAAAGGCCG-3'and 5'-AGAGAGACCCGGGACGTCCACATATACCTGCC-3'. The dC13/dG13 linker DNA was made by annealing the oligonucleotides 5'--GATCCCCCCCCCCCCCGATATC-3' and 5'--GATCGATATCGGGGGGGGGGGG -3'. The dC13/dG13 linker, the pDNR-derived DNA fragment and the mouse IgG constant gene fragment were inserted into the Bam HI/Not I site of pGFP N1 plasmid (Clontech). The pJON-mIgG plasmid was generated from the resulting plasmid by removing the pEGFP N1-derived sequence spanning from the kanamycin gene to the pUC replication origin by PCR. The pJON-mIgK plasmid was constructed with the dC13/dG13 linker, the pDNR-derived DNA fragment and the mouse IgK constant gene fragment in the same manner. A map of the plasmids is shown in Figure 1. The DNA cassette for the mouse VH (IgH-cassette) was amplified from pJON-mIgG plasmid with the primers 5'-GGGGGGGGGGGGGGGGGATCCCGG-3' and 5'-GCCTGGTCAAGGGCTATTTCCCTGAG-3'. The DNA cassette for the mouse VL (IgK-cassette) was amplified from pJON-mIgK plasmid by PCR with the primers 5'-GGGGGGGGGGGGGGGGGATCCCGG-3' and 5'-CTGTATCCATCTTCCCACCATCCAGT-3'. The amplified cassettes were treated with Dpn I to digest the template plasmid and then purified using a S-400 spin column.

### Design and fabrication of the MAGrahd reactor tray

The MAGrahd reactor tray consists of three components: drops, magnetic beads and a super-hydrophobic film-layered tray. The upper tray plate of an Omni tray (Nunc) was cut in a rectangular shape and was fixed to a thin glass cover plate (Matsunami, No. 1, 73 mm × 118 mm, 0.1 mm thickness) instead of a plastic board. Super-hydrophobic film was fabricated by pressing the film with an aluminum master stamp that contained the positive relief features against a silicone plate. The master stamp used here was made using a standard end mill process. The fabricated film has a 24 × 8 grid of positive relief features, which capture drops on a planer surface. The grid consists of two arcs that are arranged to form a cup-shaped enclosure with diameter of 3 mm. The arcs in each body project 0.15 mm upwards from a planar surface and are separated from one another by gaps of approximately 2.5 mm in width on the upper side to allow magnetic beads to move into the cup and 1 mm in width on the bottom side to allow magnetic beads to move through the grids. Twenty-four grids are arranged in rows, whose pitch matches the pitch of standard 384-well plates. The fabricated film was bound to the inner surface of the thin glass plate. On this film, droplets were spontaneously self-centered on the generated pattern, which enabled parallel sample processing by MAGrahder.

### Robotic magnetic bead handling instrument

A SHOTMASTER 300 desktop robot was converted into MAGrahder by mounting a magnetic head. Twenty-four neodymium magnet rods (Magfine, φ1.5 mm × 20 mm 2910 Gauss) were installed on the head, whose pitch matches the pitch of standard 96-well plates. With a specially designed software program, the non-contact magnetic power transmission can be adapted to achieve optimal output.

### Synthesis of 3'-end homopolymer-tailed cDNA from single cells

Plasma cells were isolated from popliteal lymph nodes of egg albumin-immunized mice using a CD138+ plasma cell isolation kit as previously described [18]. Single plasma cells were captured with a standard micromanipulator mounted on an inverted microscope and suspended in 10 μl of lysis/binding solution (100 mM Tris HCl, pH 7.5, 500 mM LiCl, 1% lithium dodecyl sulfate, 5 mM dithiothreitol) containing 5 μg of oligo-dT magnetic beads. The preparation of 3'-end homopolymer-tailed cDNA from single plasma cells was performed by the MAGrahd method as described previously [18]. Briefly, 3 μl each of single-cell lysate containing oligo-dT magnetic beads, washing solution 1, washing solution 2, reverse transcription solution, washing solution 3, terminal transferase solution, washing solution 4 and PCR solution were dispensed onto the fabricated film of the MAGrahd reactor tray. After all the regents were spotted onto the film, cDNA synthesis and homopolymer-tailing reaction were conducted with MAGrahder by touching neodymium permanent magnet rods to the opposite side of the thin glass surface of the MAGrahd reactor tray above the drops.

### Amplification of VH and VL genes from single plasma cells

VH and VL chain genes were amplified by single cell-based 5'-RACE PCR with 3'-end homopolymer-tailed cDNAs as templates. Briefly, the first round of PCR was performed with a dC13 forward primer (5'-CGGTACCGCGGGCCCGGGATCCCCCCCCCCCCCDN-3') and a mixture of reverse primers (P0) specific for the respective IgG (5'-ACCYTGCATTTGAACTCCTTGCC-3') and IgK (5'-ACTGCCATCAATCTTCCACTTGACA-3') constant regions. PCR was performed using PrimeStar DNA polymerase in 1× PrimeStar GC buffer with the BIO-RAD MyCycler (35 cycles with denaturation at 95°C for 30 s, annealing and strand elongation at 68°C for 90 s and a final extension at 72°C for 3 minutes). The resulting PCR mixtures were next diluted at a 1:10 ratio with water, and 1 μl of each was used for the second round of PCR. The second round of PCR was performed with a forward primer (P1) that annealed to the dC13 forward primer used in the first PCR and the respective nested reverse primer (P2) specific for the IgG (5'-CTGGACAGGGATCCAGAGTTCCA-3') or IgK (5'-ACTGAGGCACCTCCAGATGTTAACT-3') constant regions.

### Preparation of DNA fragments for pilot experiment

A mock DNA sequence was amplified by PCR from pEGFP-N1 (Takara-Clontech) with the primers 5'-CTTCGAATTCTGCAGTCGACGGTACCGCGGGCCGGGGTGGTGCCCATCCTGG -3' and 5'-CTGGACAGGGATCCAGAGTTCCATGCCGAGAGTGATCCCG -3' (underlined sequences are homologous to the GFP gene). The mock DNA sequence (727 bp) consisted of the upper primer-derived sequence (P1 region), a portion of the GFP gene sequence (712-1372 of nucleotide accession number: U55762), and the lower primer-derived sequence (P2 region). The mock DNA was used as a nonspecifically amplified DNA sequence for the pilot experiment. The mouse V gene fragment consisted of the upper primer-derived sequence (P1 region), the poly dC/dG sequence (T1 region), the mouse IgG V region, part of IgG constant region (T2 region) (560-600 of nucleotide accession number: AB097849) and the lower primer-derived sequence (P2 region) (601-623 of nucleotide accession number: AB097849). The amplified DNA fragments were purified using a spin column. A schematic illustration of the DNA fragment and Ig-cassettes is found in Figure 2A.

### TS-jPCR

After the second round of PCR, 1 μl of the PCR solution was mixed with 1 μl of TdT solution (1 × PrimeStar GC buffer containing 0.2 mM of each dNTP and 2 units of TdT) and incubated at 37°C for 30 min for 3'-end random nucleotide tailing. TdT was heat inactivated at 94°C for 5 min.

The random nucleotide-tailed PCR products (50-100 ng) were mixed with 20 μl of PCR mixture containing 10 ng of the IgH- or IgK-cassette, 0.2 mM of each dNTP, 1.25 U of PrimeStar DNA polymerase and 400 nM of the P3 and P4 PCR primers (5'-AGAGAAACCGTCTATCAGGGCGATGGC-3' and 5'-AGAGACCCTTTGACGTTGGAGTCCACG-3') in 1× PrimeStar GC buffer. TS-jPCR was performed for 5 cycles with denaturation at 95°C for 30 s and annealing and strand elongation at 70°C for 240 s, followed by 25-30 cycles with denaturation at 95°C for 30 s, primer annealing at 60°C for 30 s and primer extension at 72°C for 1 min.

### Expression of recombinant mouse monoclonal antibodies

The unpurified TS-jPCR products (1 μl each) of paired IgH- and IgL-expression constructs were co-transfected with FuGENE HD Transfection Reagent (Roche) into 293FT cells grown in 96-well culture dishes without the risk of endotoxin contamination derived from the plasmid purification step out of bacteria. Two days after transfection, the cell culture supernatants were analyzed for the secretion of recombinant antibodies. Antibody concentration and antibody reactivity were determined by a sandwich enzyme-linked immunosorbent assay (ELISA) as described previously [18].

### Sequence Analysis

The nucleotide sequence was determined using an Applied Biosystems 373 DNA sequencer with a BigDye Terminator v3.1 Cycle Sequence Kit (Applied Biosystems). The alignment of the V-(D)-J sequence was performed using IMGT/V-QUEST http://imgt.cines.fr/IMGT_vquest/share/textes/[20].

## List of abbreviations used

PCR: polymerase chain reaction; TS-jPCR: target-selective joint PCR; GFP: green fluorescent protein; V: immunoglobulin variable; VL: immunoglobulin light chain variable; VH: immunoglobulin heavy chain variable; 5'-RACE PCR: rapid amplification of 5'cDNA ends polymerase chain reaction; ELISA: enzyme-linked immunosorbent assay; IgG: immunoglobulin gamma; IgK: immunoglobulin kappa; TdT: terminal deoxynucleotidyl transferase, RLU: relative light unit.

## Authors' contributions

MY performed the experiments and data analysis described in this study. NK designed the experiments and drafted the manuscript. MI organized the experiments. All authors read and approved the final manuscript.
